# Multifeature Fusion Attention Network for Suicide Risk Assessment Based on Social Media: Algorithm Development and Validation

**DOI:** 10.2196/28227

**Published:** 2021-07-09

**Authors:** Jiacheng Li, Shaowu Zhang, Yijia Zhang, Hongfei Lin, Jian Wang

**Affiliations:** 1 College of Computer Science and Technology Dalian University of Technology Dalian China

**Keywords:** suicide risk assessment, social media, infodemiology, attention mechanism, neural networks

## Abstract

**Background:**

Suicide has become the fifth leading cause of death worldwide. With development of the internet, social media has become an imperative source for studying psychological illnesses such as depression and suicide. Many methods have been proposed for suicide risk assessment. However, most of the existing methods cannot grasp the key information of the text. To solve this problem, we propose an efficient method to extract the core information from social media posts for suicide risk assessment.

**Objective:**

We developed a multifeature fusion recurrent attention model for suicide risk assessment.

**Methods:**

We used the bidirectional long short-term memory network to create the text representation with context information from social media posts. We further introduced a self-attention mechanism to extract the core information. We then fused linguistic features to improve our model.

**Results:**

We evaluated our model on the dataset delivered by the Computational Linguistics and Clinical Psychology 2019 shared task. The experimental results showed that our model improves the risk-F1, urgent-F1, and existence-F1 by 3.3%, 0.9%, and 3.7%, respectively.

**Conclusions:**

We found that bidirectional long short-term memory performs well for long text representation, and the attention mechanism can identify the key information in the text. The external features can complete the semantic information lost by the neural network during feature extraction and further improve the performance of the model. The experimental results showed that our model performs better than the state-of-the-art method. Our work has theoretical and practical value for suicidal risk assessment.

## Introduction

The World Health Organization’s statistical report showed that millions of people choose to commit suicide every year, and even more people are preparing to implement suicide. In 2016, 21.2 in 100,000 people chose to commit suicide worldwide. Moreover, approximately 300,000 people commit suicide in China every year, and the number of suicide attempts is close to 200,000. Suicide has become the fifth leading cause of death worldwide [1]. The traditional suicide risk assessment method is only dependent on the diagnosis of psychologists, which has great deficiencies with respect to inefficiency and coverage. With development of the internet, social media platforms such as Twitter, Sina Weibo, and WeChat Moments have developed rapidly in recent years. Social media has gradually become an integral part of our lives. People communicate with each other through social media, and use it as a platform to express their emotions and share their opinions, including suicidal social media posters who use these platforms to express their feelings. It is estimated that 68% of the people who use social media are 10 to 30 years old. Since the high-risk population for suicide is concentrated in the age group of 15 to 29 years, there is considerable overlap between these cohorts [2]. This means that social media is an important data source for studying psychological illnesses such as depression and suicide.

In recent years, text mining based on social media and its psychologically related submedia has become a hot topic in computational linguistics, which provides new research methods for social media–oriented suicide risk assessment. Many scholars have assessed suicide risk by extracting psychological features from texts. For example, Huang et al [3] proposed a method to detect the suicide risk of social media users by identifying mental vocabulary. Zhang et al [4] proposed a method of using linguistic features to assess suicide risk. However, this method has poor detection accuracy and generalization ability, leading to the development of machine learning–based approaches to tackle the task of suicide risk assessment. Kumar et al [5] analyzed the posting activities of posters on the SuicideWatch subreddit that followed celebrity suicide news. They proposed a suicide risk assessment method based on the Werther effect and latent Dirichlet allocation [6] model. De Choudhury et al [7] analyzed the transition process of user tweets from mental health content to suicide content. They proposed a statistical method based on propensity score matching to detect the user’s suicidal intent. Bittar et al [8] proposed a method to detect suicide risk using machine learning for electronic health records. Ji et al [9] proposed a new data protection scheme and average difference reduction optimization strategy (AvgDiffLDP) to improve the machine learning model. In addition to machine learning–based methods, deep learning–based methods also have shown good performance in text classification. Shing et al [10] proposed a convolutional neural network (CNN) fused with external dictionary features to detect suicide risk. Mohammadi et al [11] proposed a multichannel classification model including a CNN and recurrent neural network (RNN).

It is necessary to judge the text from different angles when assessing the suicide risk of posts. However, it is difficult for a single model to fully capture the semantic information of the text. Therefore, inspired by previous work [10,11], we here propose a multifeature fusion recurrent attention model for the social media–oriented suicidal risk assessment task. The attention model is used to capture the semantic information in the text and merge it with other external features to better assess the effect.

The main contributions of this paper are divided into the following aspects. First, we propose a recurrent attention model. Using this model to represent the text can extract the core semantic information of the text. We further introduce a distribution loss function to reduce the impact of uneven data distribution.

Second, we fuse external features based on neural networks. These external features are valuable in suicide risk assessment and can further improve the performance of our model.

Finally, experimental results showed that our model achieved state-of-the-art performance on the suicide risk assessment dataset, demonstrating that the model has excellent performance and good practical value.

## Methods

### Multifeature Fusion Recurrent Attention Network

**Figure 1:**
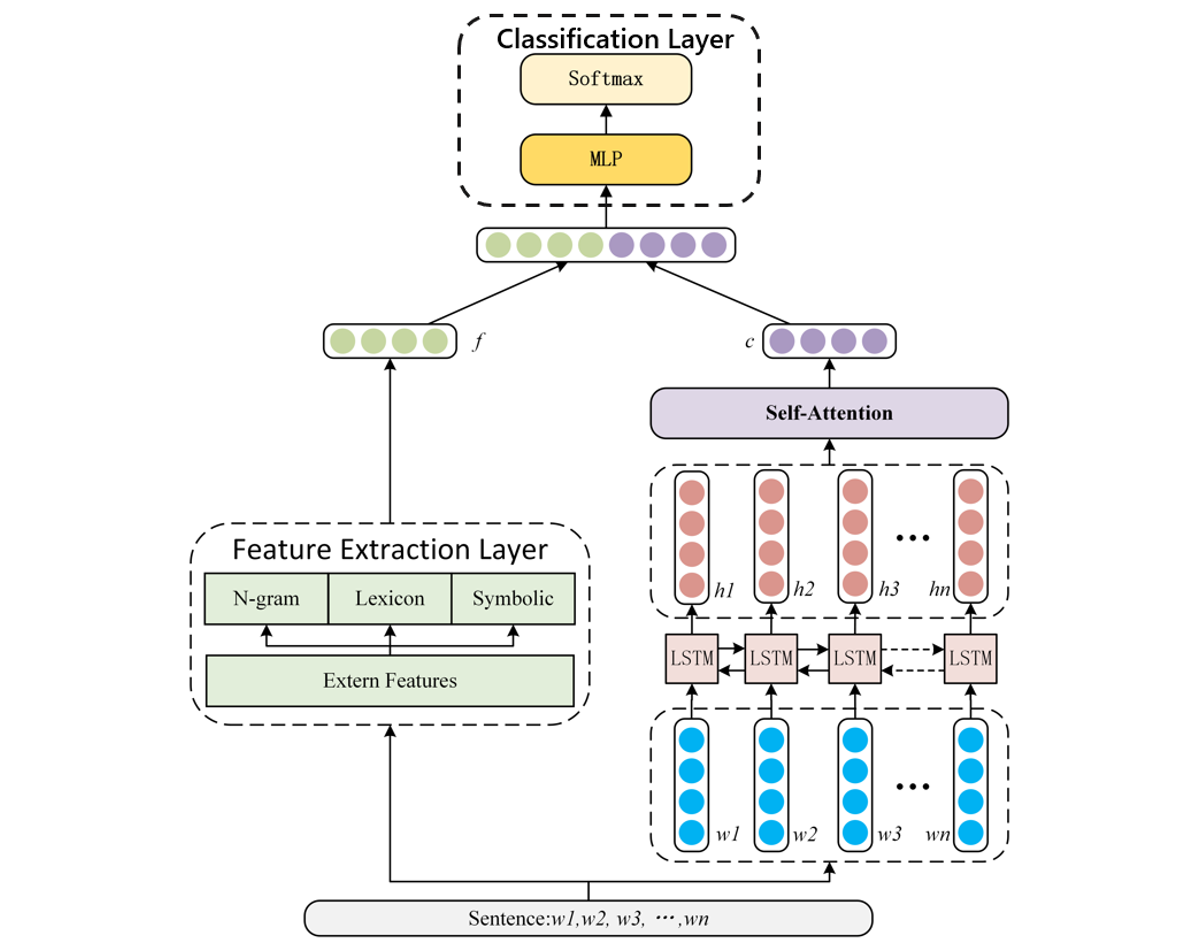
Architecture of the multifeature fusion recurrent attention network. LSTM: long short-term memory; MLP: multilayer perceptron.

The multifeature fusion recurrent attention method proposed in this paper consists of four parts. The framework of our model is shown in Figure 1. The first part of the model uses a long short-term memory network (LSTM) to obtain the text representation *T*, which has an attention weight *α* in the second part of the model-attention mechanism. The third part of the model is the feature extraction layer, which is used to capture features in the post that are difficult to be extracted by the neural network. The model then fuses the external feature vector with the attention vector to assess suicide risk.

### LSTM Network

The LSTM network was proposed by Hochreiter et al [12], which is a variant of the RNN. LSTM introduces a “gate layer” to control neurons to update information, increasing the ability to avoid long-distance dependency problems. LSTM further solves the gradient explosion and gradient disappearance of an RNN when training long text. Therefore, LSTM is the best choice for solving long text classification tasks. The algorithm process of LSTM is as follows:

*f_k_*=*σ*(*W^f^x_k_* + *V^f^h_k–1_* + *b^if^*) **(1)**

*i_k_*=*σ*(*i^f^x_k_* + *V^i^h_k–1_* + *b^f^*) **(2)**

*o_k_*=*σ*(*W^o^x_k_* + *V^o^h_k–1_* + *b^o^*) **(3)**

*c′_k_*=*tanh*(*W^c^x_k_* + *V^c^h_k–1_* + *b^c^*) **(4)**

*c′_k_*=*f_k_* ⊙ *c_k–1_* + *i_k_* ⊙ *c′_k_***(5)**

*h_k_=o_k_* ⊙ *tanh* (*c_k_*) **(6)**

where *σ* represents the sigmoid function and ⊙ represents the element-wise multiplication of two vectors. If an input sequence is *X*=[*x_1_, x_2_, x_3_,…,x_N_*] for the input *x_k_*(1≤*k*≤*N*) of each position, LSTM needs three steps to output the hidden state *h_k_*. In the first step, the forget gate *sigmoid* function decides whether the memory cell *c_k_* needs to forget information based on the hidden state *h_k–1_* of the previous position and input *x_k_*. The next step is to decide what information the memory cell needs to update, and this step can be divided into two parts. First, the input gate *sigmoid* function determines whether the memory cell needs to update information. Then, the *tanh* function will generate a new candidate value *c′_k_*. The new state of the memory cell will be updated under the joint action of the forgetting gate and input gate. In the last step, the hidden state of this position is limited between 0 and 1 under the action of the *tanh* function, and the output gate sigmoid function decides whether the neuron needs to output.

LSTM can obtain the information of the current position through the above steps, but the text below is also essential. In the bidirectional LSTM (BiLSTM), the forward LSTM can extract the above information and the backward LSTM can extract the following information. The BiLSTM combines the above hidden state and the below hidden state in the same position to create a new hidden state, which can obtain more context information. The hidden state *h_k_* of the BiLSTM is shown in Equation 9.


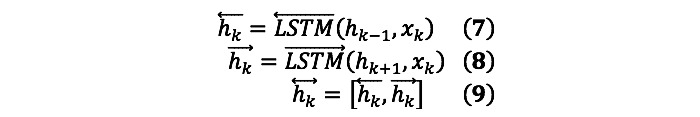


### Self-Attention Layer

In a sentence, there are only a few words that can represent the semantic information of the entire sentence. If the model treats every word the same way, the learning ability of the model will be wasted, which will reduce the efficiency of the model. Therefore, we introduce the attention mechanism to this process. This adds an attention weight to each word in the text so that the model will pay more attention to words with higher weights. The attention mechanism has achieved excellent performance in natural language processing tasks owing to its advantages of fewer parameters, faster model training, and stronger interpretability [11].

For the hidden state 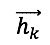 from the BiLSTM, the calculation process to obtain the attention weight 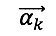 is as follows:


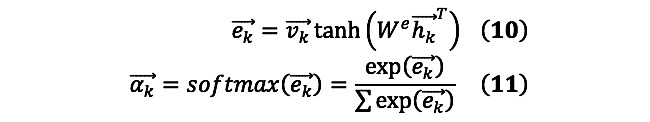


where 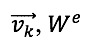 are trainable parameters and 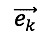 is the attention score of the input hidden state 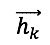. Normalization of 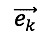 is the softmax function that can provide the attention weight of the input. The vector representation of the entire sentence can then be calculated by Equation 12:


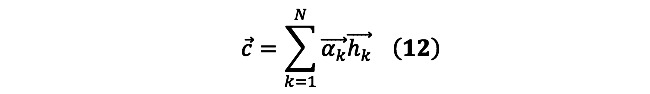


### Feature Extraction Layer

The neural network focuses on the semantic information of the text, but there are other linguistic features in the text that can help to assess suicide risk. We set up three sets of linguistic features: n-gram features, lexicon-based features, and symbolic features.

For n-gram features, we used bigram and trigram linguistic models as features, and we used term frequency-inverse document frequency (TF-IDF) weights to calculate the feature values. However, the feature matrix is very sparse, and therefore we used nonnegative matrix factorization [13] to reduce the dimension to 50.

For lexicon-based features, since a sentiment word represents the sentiment tendency of the entire text, we introduced the NRC [14] dictionary to capture the posters’ emotions. We separately counted the number of emotional words representing positive emotions, negative emotions, sadness, anger, despair, and fear in a post, and the length of the post. We combined these statistics as a lexicon-based feature vector.

For symbolic features, Stirman et al [15] proposed that suicidal people are self-oriented and they frequently use first-person pronouns. Yang et al [16] proposed that suicidal people frequently use rhetorical rhetoric to emphasize their emotions consciously. In social media posts, emojis are also used to express emotions. Therefore, we counted the number of first-person pronouns (eg, “I,” “me,” “mine,” “myself”), question marks, and emojis in posts as symbolic features.

### Classification Layer

The classification layer used in this study consisted of two parts: a multilayer perceptron and softmax layer. The multilayer perceptron produces classification results and the classification probability is normalized by the softmax layer. We also used the distribution loss function to train the model. Owing to the small number of samples in the dataset, we introduced *L_2_* regularization to reduce the overfitting problem of the model.


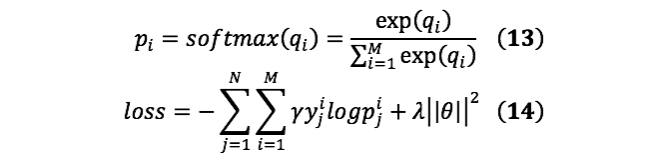


where *N* is the total number of training data, *M* is the number of categories, and *q_i_* and 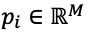 represent the classification result and classification probability, respectively. In Equation 14, *y^i^_j_* is the ground truth, *λ* is the coefficient of the *L_2_* regularization term, and *θ* is a hyperparameter. In particular, we introduced the distribution weight γ in the loss function, which is a trainable parameter [17]. Categories with more training data have smaller weights. The distribution loss function can reduce the impact of an uneven data distribution.

### Experimental Settings

Before the experiment, we set the initial parameters based on previous modeling experience. We tuned the model parameters on the development set and achieved the best results. We used Adam to optimize the model. The parameters of the optimal model are shown in Table 1.

**Table 1:** Hyperparameter settings.

Hyperparameters	Optimal value
Word embedding dimension	300
BiLSTM^a^ hidden units	200
Learning rate	0.2
Dropout rate	0.5
L_2_ regularization weight	10^–5^

^a^BiLSTM: bidirectional long short-term memory.

## Results

### Dataset

The suicide risk assessment dataset was released by the Computational Linguistics and Clinical Psychology (CLPsych) 2019 shared task. The goal of CLPsych 2019 was to assess users’ suicide risk based on their posts. The dataset constructed by Shin et al [10] in 2018 consists of posts published on the Reddit social media platform between 2005 and 2015. To protect users’ privacy, their personal information was replaced by a user ID.

This paper is based on CLPsych-2019 task A (“From Keyboard to Clinic”). Texts used in our dataset were all derived from posts with varying degrees of suicide risk on the SuicideWatch subreddit. The CLPsych dataset was broken down to include 57,016 posts in the training set and 9611 posts in the test set, all from the SuicideWatch subreddit. Among them, the proportion of samples in each category was close to 1:1:1:1. The shortest sentence contained 14 words and the longest sentence contained 486 words. We defined the three following assessment methods to better assess the suicide risk and increase the practicality of the model: (1) suicide risk (risk), which has the same requirements of the CLPsych share task, divided into four classes *a, b, c, d* from low to high; (2) suicide existence (existence), which is an indicator used to judge whether the poster has a suicidal intention so that the posts can be divided into two levels of exist versus not exist, with the latter indicating a shallow suicide risk (*class a*), and they are not likely to commit suicide in the near future; (3) suicide urgency (urgency), in which the post is divided into two levels of urgent versus not urgent according to the suicide risk, with the urgent level (classes *a, b*) indicating that the user needs psychological assistance urgently.

Table 2 shows the postassessment results obtained under the different suicide risk assessment methods.

**Table 2:** Example posts from the SuicideWatch subreddit.

Post	Risk	Existence	Urgency
A nihilist teetering on edge. Things were good before I came into being	a	Not exist	Not urgent
Has anyone attempted suicide and failed and then felt guilty for being incompetent?	b	Exist	Not urgent
Just sitting on a bench, waiting and thinking. I don’t want to, but it feels like the best option.	c	Exist	Urgent
Tell me how to commit suicide painlessly.	d	Exist	Urgent

### Evaluation Metrics

In the experiments, the performance of our model was evaluated by the macroaverage *F_1_* score. The verification method was as follows:

*P*=*TP*/*TP*+*FP***(15)**

*R*=*TP*/*TP*+*FN***(16)**

*F_1_*=2×*P*×*R*/*P*+*R***(17)**

where *P* and *R* are precision and recall, respectively. TP, FN, and FP represent the true positive, false negative, and false positive predictions, respectively. The *F_1_* score is a harmonic average of precision and recall.

### Comparison With Baseline

To compare the performance of different models in the suicide assessment task, we tested different classification models on the training set. The experimental results are shown in Table 3.

The inputs of the above models are all 300-dimensional Glove word embedding vectors. As shown in Table 3, the performance of the deep learning–based models was better than that of the machine learning–based models. The results of the LSTM and BiLSTM were also better than those of the CNN. In particular, LSTM was better than CNN for long text processing, and the performance of BiLSTM was better than that of LSTM. This shows that BiLSTM can capture more contextual semantic information. The results of the ensemble models were significantly better than those of the single models. In addition, different models showed different capabilities of semantic information extraction, and the combination of different models can supplement the missing semantic information of a single model. The result of the BiLSTM+Attention model was better than that of the BiLSTM+CNN model. This assessment demonstrated that our introduced attention mechanism is more suitable for this task.

**Table 3:** Experimental results of classification models.

Models	Risk-F_1_	Existence- F_1_	Urgency -F_1_
SVM^a^	0.296	0.793	0.716
CNN^b^	0.336	0.834	0.742
LSTM^c^	0.397	0.862	0.766
BiLSTM^d^	0.404	0.863	0.774
BiLSTM+CNN	0.423	0.872	0.789
BiLSTM+Attention (proposed model)	0.448	0.887	0.796

^a^SVM: support vector machine.

^b^CNN: convolutional neural network.

^c^LSTM: long short-term memory.

^d^BiLSTM: bidirectional long short-term memory.

### Comparison of Different Input Features

In addition to using the deep learning–based model, we also set up three sets of linguistic features: n-gram features, lexicon-based features, and symbolic features. To test the influence of different features on the suicide risk assessment task, we set up 6 sets of comparative experiments. We separately recorded the experimental results of a support vector machine (SVM) model. The experimental results are shown in Table 4.

The *risk-F_1_* score using TF-IDF features was 0.257. The performance of the n-gram–based method was better than that of TF-IDF. The results of the trigram were better than those of the bigram. Using lexicon features had the most significant improvement on the results, whereas the symbolic features improved the performance to a lesser extent. Concatenating all feature vectors showed that using ensemble features was the best choice for our task, with a *risk-F_1_* score of 0.284.

We further compared the effects of embedding methods on the experimental results. The pretraining language model bidirectional encoder representations from transformers (BERT) can also be used for classification tasks alone. We compared the pretraining language model BERT with the BiLSTM and BiLSTM+Attention models, which showed excellent performance on our task. We used word2vec word embedding [18], Glove word embedding [19], and BERT embedding as the input of the model. The experimental results are shown in Table 5.

The result improved slightly after adding LSTM. Using the pretrained language model BERT resulted in better performance than using the word embedding model. We also concatenated ensemble features at the classification layer, which further improved the performance of the model.

**Table 4:** Experimental results of different features for support vector machine models.

Input	Risk-F_1_	Existence- F_1_	Urgency -F_1_
TF-IDF^a^	0.257	0.783	0.691
Bigram+TF-IDF	0.271	0.802	0.712
Trigram+TF-IDF	0.276	0.798	0.709
Lexicon+TF-IDF	0.282	0.826	0.721
Symbolic+TF-IDF	0.254	0.784	0.684
n-gram+lexicon+symbolic+TF-IDF	0.284	0.835	0.724

^a^TF-IDF: term frequency-inverse document frequency.

**Table 5:** Experimental results of deep learning–based models.

Models and input			Risk-F_1_		Existence- F_1_	Urgency -F_1_
BERT^a^			0.467		0.889	0.861
**BiLSTM^b^**						
	Word2vec	0.404		0.863		0.774
	Glove	0.412		0.861		0.793
	BERT	0.474		0.914		0.857
	BERT+Features	0.481		0.923		0.863
**BiLSTM+Attention**						
	Word2ve	0.448		0.887		0.796
	Glove	0.456		0.891		0.787
	BERT	0.507		0.915		0.863
	BERT+Features	0.514		0.931		0.876

^a^BERT: bidirectional encoder representations from transformers.

^b^BiLSTM: bidirectional long short-term memory.

### Comparison With Other Existing Models

We compared our model with the methods of other teams in the CLPsych 2019 shared task, demonstrating that our model achieved the best results. The risk-*F_1_*, urgent-*F_1_*, and existing-*F_1_* all reached the highest levels with our proposed model (Table 6).

**Table 6:** Experimental results of existing methods.

Models	Risk-*F*_1_	Existence-*F*_1_	Urgency-*F*_1_
Mohammadi et al [11]	0.481	0.922	0.776
Matero et al [20]	0.459	0.842	0.839
Bitew et al [21]	0.445	0.852	0.789
Iserman et al [22]	0.402	0.902	0.844
Allen et al [23]	0.373	0.876	0.773
González Hevia et al [24]	0.312	0.897	0.821
Multifeature fusion recurrent attention (this study)	0.514 (+0.033)	0.931 (+0.009)	0.876 (+0.037)

Mohammadi et al [11] proposed an ensemble method including 8 neural submodels to extract neural features. They then used the SVM classifier to classify the neural feature vector. They achieved a risk-*F_1_* score of 0.481 and an existence-*F_1_* score of 0.922 (the highest result in CLPsych 2019). González Hevia et al [24] also proposed an ensemble method combined with the result of the SVM classifier and a pretrained RNN. Marero et al [20] proposed multilevel dual-context language and BERT using the deep attention model to extract dual-context information. Their model was also fused with linguistic features and achieved the highest urgency-*F_1_* score of 0.839. Bitew et al [21] proposed a machine learning–based method, and integrated the logistic regression classifier and the linear SVM classifier. Iserman et al [22] proposed a simple recursive partitioning model with lexicon features. Similarly, Allen et al [23] used CNN and Linguistic Inquiry and Word Count [25] features to assess suicide risk.

### Attention Visualization and Error Analysis

**Figure 2:**
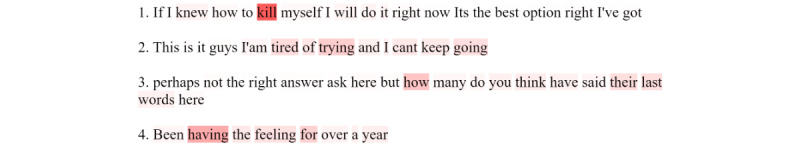
Examples of attention visualization.

To analyze the effectiveness of the attention mechanism, we extracted the attention weight of the self-attention layer and visualized it with text. The attention visualization results are shown in Figure 2; a deeper color indicates a larger attention weight for the word.

Among the four posts shown in Figure 2, the first two posts are classified into the right class by the model, whereas the last two posts are classified into the wrong category. As shown in the first post, “kill” has the largest weight, which is the core word of this post, and the model also pays attention to “knew” and “do it now.” The model then classified this post into *class d* (high suicide risk). In the second post, the model focused on “tired of trying” and “can’t keep going.” This shows that the model pays attention to words that represent the emotion of the poster. This post lacks the terms associated with high suicide risk, and therefore the model classified this post into *class c*.

In the third post (*class b*)*,* the model focused on the terms “how” and “their last words.” However, the model did not learn that the subject of “last words” was “they” instead of the poster, and therefore mistakenly classified the post into *class d*. In the fourth post (*class a*), the model focused on “having,” “feeling,” and “for a year,” and mistakenly believed that this post reflects a high suicide risk. This is because we found that “feeling” is often associated with words that express negative emotions in the training set. Therefore, we believe that the accuracy can be improved by fusing external features.

## Discussion

### Principal Findings

The results of n-gram features based on TF-IDF weights were better than those obtained using TF-IDF features, which cannot capture the word order information in the text. However, the results of trigram features were inferior to those of bigram features. This shows that although n-gram features can capture the word order information, if multiple features are extracted, the feature vectors will be sparse and reduce the performance of the model. In the experiment, using dictionary features improved the model’s performance significantly. This demonstrates that the emotional tendency of a text can be represented by the limited number of emotional words in the text. The use of symbolic features showed only minor improvements on performance, indicating that punctuation in the text can also express part of the semantic information.

Our model uses the BERT pretraining model as input. The pretrain word vectors represent the semantic information of words, making up the missing information of word embedding models.

The experimental results further showed that BiLSTM performs well in extended text classification. BiLSTM can capture the semantic information of the context in the text and solve long-distance dependence in text processing. After adding the attention mechanism, the performance of the model was further improved. This shows that the attention mechanism can effectively make the model pay attention to the core semantic features of a text.

### Conclusions

This paper proposes a multifeature fusion recurrent attention network to assess the suicide risk of SuicideWatch subreddit posts. Our model uses the BERT pretrained language model as input, which can create a more precise text representation than the word embedding model. The BiLSTM in the model can capture long-distance dependence and dual-content information. The self-attention mechanism can make the model focus on the core information of the post. The model achieved the best performance on the experimental dataset. Moreover, we introduced n-gram features, lexicon features, and symbolic features, which make up the missing information in the feature extraction of the recurrent attention network, thereby improving the accuracy of the model.

In our future work, we will introduce the personality characteristics of the posters and other social media attributes of the posters for further improving suicide risk assessment.

## Abbreviations

BERT: bidirectional encoder representations from transformers
BiLSTM: bidirectional long short-term memory network
CLPsych: Computational Linguistics and Clinical Psychology
CNN: convolutional neural network
LSTM: long short-term memory network
RNN: recurrent neural network
SVM: support vector machine
TF-IDF: term frequency-inverse document frequencies
